# Feedback Artificial Shuffled Shepherd Optimization-Based Deep Maxout Network for Human Emotion Recognition Using EEG Signals

**DOI:** 10.1155/2022/3749413

**Published:** 2022-01-21

**Authors:** K. S. Bhanumathi, D. Jayadevappa, Satish Tunga

**Affiliations:** ^1^Department of Electronics and Instrumentation Engineering, JSS Academy of Technical Education, Bengaluru, VTU, India; ^2^Department of Electronics & Telecommunication Engineering, Ramaiah Institute of Technology, Bengaluru, India

## Abstract

Emotion recognition is very important for the humans in order to enhance the self-awareness and react correctly to the actions around them. Based on the complication and series of emotions, EEG-enabled emotion recognition is still a difficult issue. Hence, an effective human recognition approach is designed using the proposed feedback artificial shuffled shepherd optimization- (FASSO-) based deep maxout network (DMN) for recognizing emotions using EEG signals. The proposed technique incorporates feedback artificial tree (FAT) algorithm and shuffled shepherd optimization algorithm (SSOA). Here, median filter is used for preprocessing to remove the noise present in the EEG signals. The features, like DWT, spectral flatness, logarithmic band power, fluctuation index, spectral decrease, spectral roll-off, and relative energy, are extracted to perform further processing. Based on the data augmented results, emotion recognition can be accomplished using the DMN, where the training process of the DMN is performed using the proposed FASSO method. Furthermore, the experimental results and performance analysis of the proposed algorithm provide efficient performance with respect to accuracy, specificity, and sensitivity with the maximal values of 0.889, 0.89, and 0.886, respectively.

## 1. Introduction

Electroencephalography (EEG) is a composite time series data, and it is considered as a noninvasive technique such that the experts are utilizing it often in numerous applications. Emotion recognition is an affective computing procedure, which includes studies of how emotions are processed and recognized by computers. Emotions support humans in daily life for reasoning, planning, decision-making, and several human mental tasks. A psychologically balanced individual with positive emotions led a successful life when compared to an emotionally unbalanced individual. Besides, negative emotions may harm a person's decision-making skills and health. Moreover, emotion recognition has numerous applications in various fields, such as the entertainment industry, e-learning, adaptive games, adaptive advertisement, and an emotion-based music player. Various clinical analysts utilize EEG signals for recognizing emotions as the EEG cap is a moveable device in such a way that it is suitable and can be used in different emotion recognition applications [1]. Emotion models can be generally categorized into two different types, namely, discrete and dimensional models. According to Ekman's theory, emotions are classified into discrete entities, such as anger, fear, happiness, disgust, and surprise [2]. The latter illustrates emotions by means of underlying dimensions, such as valence, dominance, and arousal [3], where the emotions can be measured from passive to active, unpleasant to pleasant, and submissive to dominant. EEG determines the voltage variations from the cortex regions in the brain to disclose significant information regarding the different mental states of human emotions [4]. For instance, larger relative left frontal EEG action has been estimated when experiencing positive emotions. The voltage variations in various regions of brain are computed by the electrodes connected to scalp such that every electrode acquires EEG signals in a single channel [5].

Emotion recognition concentrates on recognizing the emotions of humans concerning different modalities, like body language, audio-visual expressions, and physiological signals. The physiological signals, like electrocardiogram (ECG), electroencephalogram (EEG), and electromyography (EMG), benefit from being intricate to disguise or hide when compared to various other modalities. EEG-driven emotion recognition has recently gained significant attraction towards various research fields and applications because of the progressive development of simpler, inexpensive, and noninvasive EEG recording devices. Emotion is a basic psychological condition that can have an effect on human perception, human cognition, and rational decision-making. Automatic recognition of human emotions has recently achieved progressive growth with respect to rapid improvement of brain-computer interaction (BCI). As the emotions are detected from the movement of the body and facial expression, EEG is considered as the efficient way to find the emotions of the human as the EEG signals compute and record the electrical activities from various regions of the brain [6, 7]. The recognition of emotions can be carried out by speech, body posture, facial expressions, physiological actions, etc. These methods are based on outwardly expressed emotions, which cannot measure the inner feelings. Meanwhile, EEG-driven signals reveal this secret data and offer various emotion-based patterns [8, 9]. Therefore, a variety of techniques based on the feature extraction were developed for measuring the EEG signal information. These features can measure the nonlinearity and underlying difficulty of the EEG signals [10, 11]. Recently, deep learning approaches facilitate automatic feature extraction and selection, and these approaches have huge impacts on signal and information processing [12]. The various deep learning techniques, namely, deep belief network (DBN), autoencoder (AE), and convolution neural network (CNN), have been efficiently utilized in processing the physiological signals and achieved significant results when compared with the conventional shallow models. Bimodal deep autoencoder (BDAE) extracts the high level representation features and was considered very significant for recognizing the emotions. Deep learning methods, such as AE or CNN, could not grab the temporal details of EEG signals. Recurrent neural network (RNN) is a deep learning algorithm such that the connections among the units generate a loop for processing the sequence data. RNN has been commonly utilized in the process of speech recognition and machine translation. Various types of RNN, like long short-term memory, were employed for processing and analyzing physiological signals. Moreover, bimodal long short-term memory (bimodal LSTM) approach was devised for recognizing the emotions with multimodal signals. EEG signals and the features related to eye movement were considered as inputs while using SEED dataset, whereas peripheral physiological signals and the EEG signals were considered while using DEAP dataset. In addition, a hybridized deep learning system incorporating LSTM and CNN was developed to extract the task-driven features, exploring interchannel correlation and combining the contextual data from the frames.

The key contribution of this research work is an effective and accurate emotion recognition approach to classify the human emotions using the proposed FASSO-based deep maxout network (DMN). The major focus is to design a proposed FASSO-based DMN for the human emotion recognition. In the first stage, the input EEG signals are acquired from the DEAP dataset and preprocessed using median filter. After that, the required features are extracted from the preprocessed signal for further processing. Besides, the extracted features are presented to the data augmentation stage, and then, the emotion recognition phase is performed using the DMN classifier. Furthermore, the training of the DMN classifier is carried out by the FASSO algorithm, which comprises both FAT and SSOA models.

## 2. Related Work

Emotion encompasses consciousness as well as cognition in all human beings and it plays a very significant role in all aspects of humans. Hence, recognition of emotion has become a very important research area. As part of the proposed work, this section reviews various emotion recognition techniques using EEG signals. This review also provides advantages, challenges, and limitations of the existing emotion recognition approaches.

Zhong et al. [5] developed a regularized graph neural networks (RGNN) for the automated emotion recognition. This technique reduced the overfitting problems, but it failed to control the imbalance among the testing sets and the training sets. Ekman and Keltner [2] proposed a firefly integrated optimization algorithm (FIOA) to strengthen the EEG-based emotion recognition. This technique significantly reduced the artificial selection of the work loads. However, this method suffers from computational complexity issues. Sharma et al. [10] designed a LSTM- (long short-term memory-) driven deep learning method for automated emotion recognition. This method did not involve any primary knowledge regarding the functional parameters. However, the major challenge lies in maximizing the processing speed while using larger datasets. Wei et al. [12] designed a simple recurrent unit (SRU) network and ensemble learning technique for the EEG-based emotion recognition. This method achieved comparatively lower computational cost. However, deep learning is highly reliant on computation control, and it utilizes higher time for the training process.

Chao and Dong [13] presented an advanced convolutional neural network (CNN) for recognizing the emotions from the multichannel EEG signals. The distinctive grouping technique of filter preserves the regional features with respect to the diverse areas, but this technique suffers from higher computational complexity. Salankar et al. [14] developed an empirical mode decomposition (EMD) for the emotion recognition based on EEG signals. This method was more effective for the medical recognition of high- and low-dominance regions in the subjects. However, this method failed to classify the states, such as Alzheimer's, depression, and epilepsy for enhanced outcomes. Yin et al. [15] introduced a graph convolutional neural networks (ECLGCNN) and LSTM for recognizing the emotions using EEG signals. The processing time of this method was low and maintains poor recognition accuracy. Pandey and Seeja [1] devised a deep CNN for recognizing the EEG emotions. Here, frontal electrodes are more effective for recognizing the emotions when compared to all other electrodes. However, this technique failed to apply attention mechanisms on various brain regions in order to achieve effective performance results.

Maheshwari et al. [16] proposed the deep CNN architecture; it has eight convolutions, three average pooling, four batch normalization, three spatial dropouts, two dropouts, one global average pooling, and three dense layers. It is validated using three publicly available databases: DEAP. But still it suffers with poor accuracy in classifying various emotions. Hector et al. [17] work, architect, design, implement, and test a handcrafted, hardware convolutional neural network, named BioCNN, optimized for EEG-based emotion detection and other biomedical applications. The EEG signals are generated using a low-cost, off-the-shelf device, namely, Emotiv Epoc+, and then denoised and preprocessed ahead of their use by BioCNN. For training and testing, BioCNN uses three repositories of emotion classification datasets, including the publicly available DEAP and DREAMER datasets. Hu et al. [18] presented a novel convolutional layer, called the scaling layer, which can adaptively extract effective data-driven spectrogram-like features from raw EEG signals. Furthermore, it exploits convolutional kernels scaled from one data-driven pattern to expose a frequency-like dimension to address the shortcomings of prior methods requiring hand-extracted features or their approximations. This has achieved state-of-the-art results across the established DEAP and AMIGOS benchmark datasets. Liu and Fu [19] have proposed an emotion recognition by deeply learned multichannel textural and EEG features. In this work, multichannel features from the EEG signal for human emotion recognition are applied. Here, the EEG signal is generated by sound signal stimulation. Specifically, applying multichannel EEG and textual feature fusion in time domain recognizes different human emotions, where six statistical features in time domain are fused to a feature vector for emotion classification. It conducts EEG and textual-based feature extraction from both time and frequency domains. Various challenges of human emotion recognition approaches are as follows.

- SRU was developed for the emotion recognition. However, the major challenge lies in selecting the appropriate SRU network parameters, like training parameters and the total nodes based on the trial-and-error technique [12]
- FIOA offers a hybridized optimization scheme for recognizing the patterns of higher dimensionality datasets, but the experimental information used in this method are simply multiple physiological signals. Hence, the major challenge lies in using the FIOA to automatic pattern detection of medical image data [20]
- The EMD technique implemented can be valuable for medical recognition of low- and high-dominance regions in the subjects, but major challenge lies in classifying the various brain conditions, like sadness, Alzheimer's, and epilepsy [14]
- Deep CNN approach was very efficient in focusing the independent emotion recognitions with respect to classification accuracy when compared with various existing techniques. This can be enhanced by applying attention mechanisms on various regions of the brain for improving the classification accuracy [1]

Based on the literature review inference, the ECLGCNN achieved better classification accuracy, which explores only the binary categorization of emotions, such as positive or negative valence and low/high arousal. This can be made to overcome by considering ECLGCNN as a multiclassifier for effectively distinguishing the different states of emotions [15].

## 3. Proposed Method

**Figure 1:**
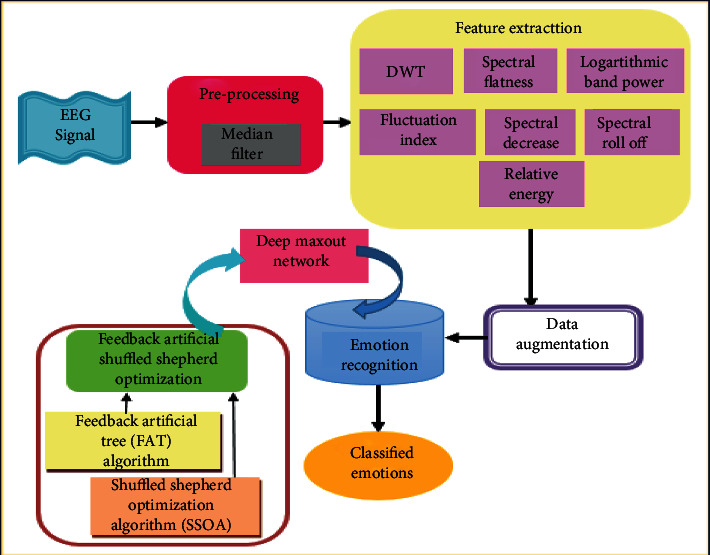
Block diagram of the proposed FASSO-driven deep maxout network for human emotion recognition.

The proposed FASSO-based deep maxout network (DMN) for the human emotion recognition mainly consists of four stages, namely, preprocessing, feature extraction, data augmentation, and finally emotion recognition. In the first stage, input EEG signals are preprocessed using the median filter to remove unwanted noise. After that, the feature extraction process is carried out in order to extract the required features, such as DWT, spectral flatness, logarithmic band power, fluctuation index, spectral decrease, spectral roll-off, and relative energy. Once the feature extraction phase is completed, the data augmentation is done by adding the noises to the original signals for generating the new samples. Finally, the emotion recognition is performed using the DMN classifier [21] for recognizing the emotions, like pride, elation, joy, satisfaction, relief, hope, interest, surprise, sadness, fear, shame, guilt, envy, disgust, contempt, and anger. In this case, the training process of the DMN classifier is carried out using the proposed FASSO approach. This approach is newly designed by the integration of FAT [22] and SSOA [23].  Figure 1 shows the schematic representation of the proposed FASSO-based DMN for human emotion recognition using EEG signals.

Let us consider a DEAP dataset with EEG signals of *G* amount of human emotions, which is given as follows:

(1) $$\begin{array}{c} Χ=\left\{J_{h};1\leq h\leq G\right\}, \end{array}$$

where *J*_*h*_ signifies the EEG signals of *h*^th^ data and *G* indicates the total human emotions. Here, the input EEG signal *J*_*h*_ is subjected as an input to the preprocessing phase in order to remove the noise from the original signals. The removal of noise is the fundamental step to enhance the input signals for further processing. Thus, median filter is used for an efficient preprocessing and the corresponding equation for preprocessing is as follows.

(2) $$\begin{array}{c} S_{2}\left(P,Q\right)={\text{median}}_{\left(J_{h}\right)\in G_{P,Q}}\left\{l\left(J_{h}\right)\right\}, \end{array}$$

where *p* and *q* are the signal information and the median filter output is denoted as *Μ*_1_.

### 3.1. Feature Extraction

Once the preprocessing is performed, the feature extraction can be done for extracting the significant features for further processing. The median filter output *M*_1_ is applied to extract features such as DWT, spectral flatness, logarithmic band power, fluctuation index, spectral decrease, spectral roll-off, and relative energy. The extracted features are explained in the following sections.

#### 3.1.1. DWT Features

DWT feature [24] is used for the signal transformation from spatial to frequency domain, and in this case, Haar wavelet transform is used. This wavelet transform extracts the information from the signals at different scales by passing the EEG signals through the low-pass and high-pass filters. Moreover, the wavelet features enable superior multiresolution capabilities and energy compaction functionalities. Thus, the size of DWT feature is in the dimension of [1 × 64] and is signified as*q*_1_.

#### 3.1.2. Spectral Flatness

Spectral flatness is utilized in digital signal processing in order to characterize the audio spectrum and is measured in decibels. It is measured by the ratio of the geometric mean to the arithmetic mean of power spectrum, and the equation is expressed as follows:

(3) $$\begin{array}{c} q_{2}=\frac{\sqrt[{M}]{\prod_{v=0}^{M-1}b\left(v\right)}}{\sum_{v=0}^{M-1}b\left(v\right)/M}=\frac{\exp\left(\left(1/M\right)\sum_{v=0}^{M-1}\ln b\left(v\right)\right)}{\left(1/M\right)\sum_{v=0}^{M-1}b\left(v\right)}, \end{array}$$

where *b*(*v*) signifies the magnitude of bin number *v*, and moreover, spectral flatness feature is in the dimension of [1 × 80] and is denoted as *q*_2_.

#### 3.1.3. Logarithmic Band Power

Logarithmic band power is computed using the logarithmic power of different EEG signal bands. Hence, this feature is measured by computing the band power frequency value, and the equation is expressed as follows:

(4) $$\begin{array}{c} q_{3}=\log\left(C_{t}\right), \end{array}$$

where log(*C*_*t*_) signifies the logarithmic band power of the preprocessed signal and the term *q*_3_ represents the logarithmic band power. The logarithmic band power feature is in the dimension of [1 × 40].

#### 3.1.4. Fluctuation Index

Fluctuation index is used for computing the characteristic value of the EEG signals, and the equation is expressed as follows:

(5) $$\begin{array}{c} q_{4}=\frac{1}{A}\overset{A}{\underset{z=1}{\sum }}\left|y\left(z+1\right)-y\left(z\right)\right|, \end{array}$$

where *A* represents the signal data points, *y*(*z*) signifies the *V* points in the signal value, and the fluctuation index feature is in the dimension of [1 × 1] and is indicated as *q*_4_.

#### 3.1.5. Spectral Decrease

The spectral decrease is used for computing the reduction in the magnitude spectrum over a time. On the other hand, spectral decrease represents the amount of spectrum decrease while emphasizing slopes of minimum frequencies, and the equation for spectral decrease is formulated as follows:

(6) $$\begin{array}{c} q_{5}=\frac{\sum_{r=u_{1}+1}^{u_{2}}\left(s_{r}-s_{r_{1}}\right)/\left(r-1\right)}{\sum_{r=u_{1}+1}^{u_{2}}s_{r}}, \end{array}$$

where the term *q*_5_ indicates the spectral decrease and the size of spectral decrease feature is in the dimension of [1 × 80], respectively.

#### 3.1.6. Spectral Roll-Off

Spectral roll-off is defined as the frequency of the signal below which a particular percentage of the overall spectral energy lies, and spectral roll-off feature is in the dimension of [1 × 80]. In addition, the term *q*_6_ specifies the spectral roll-off feature.

#### 3.1.7. Relative Energy

Relative energy is utilized for examining the variations in the EEG frequency bands, and the relative energy feature is in the dimension of [1 × 1] and is indicated as *q*_7_. Finally, the extracted features are integrated together in order to generate a feature vector output indicated as*F*.

(7) $$\begin{array}{c} F=\left\{q_{1},q_{2},q_{3},q_{4},q_{5},q_{6},q_{7}\right\}. \end{array}$$

The size of the feature vector dimension is [1280 × 347]. The extracted feature *F* is fed as an input to data augmentation phase for maximizing the emotion recognition performance effectively. In the data augmentation process, additive white Gaussian noises are added to original input signal *J*_*h*_ to generate the new samples. After that, the generated new samples are incorporated with the feature vector output *F* extracted from the feature extraction phase to obtain the final feature output with the size of [2560 × 347]. The data augmentation output is denoted as *D*_1_.

### 3.2. Emotion Recognition

**Figure 2:**
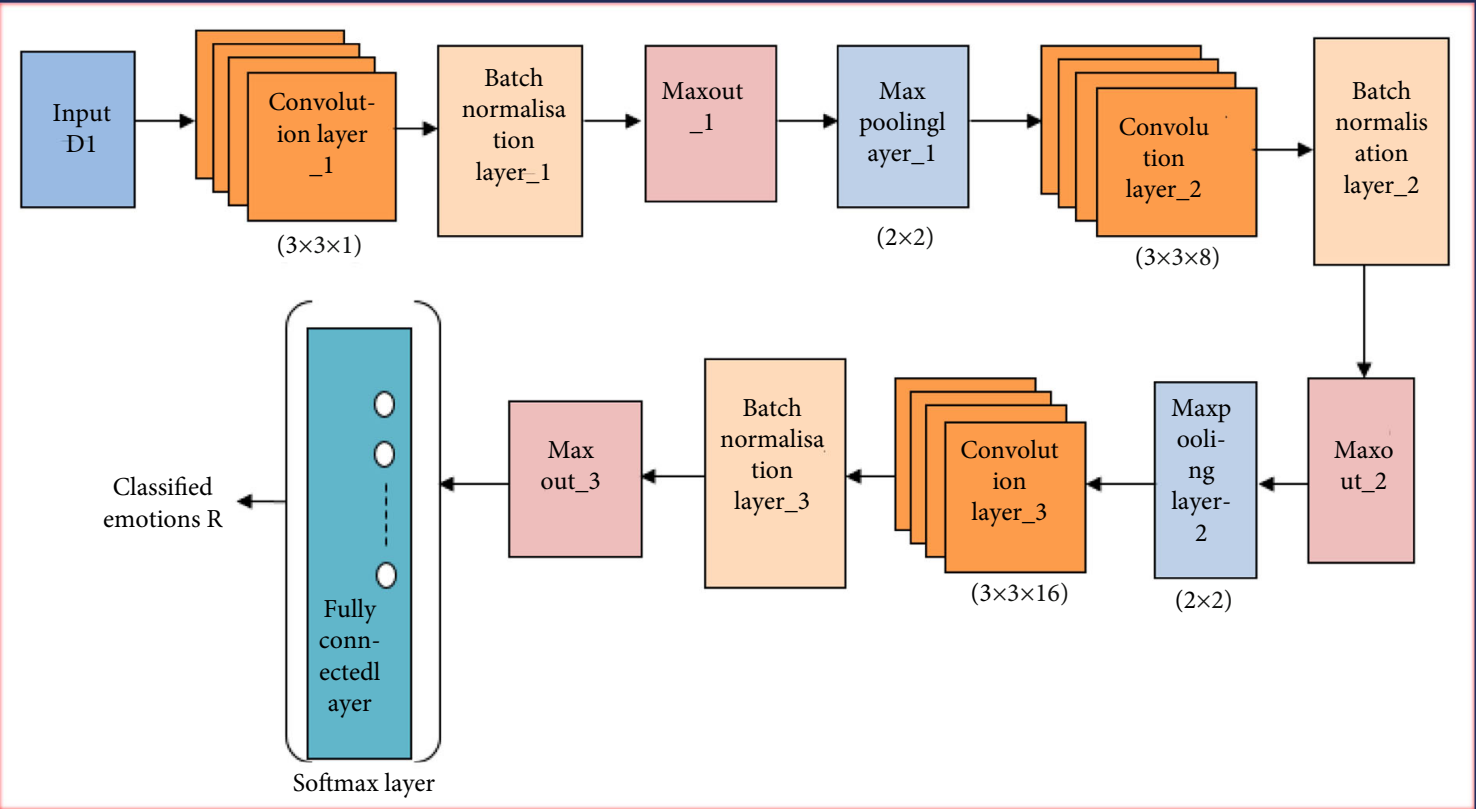
Architecture of the deep maxout network (DMN) classifier.

Once the data augmentation is performed, emotion recognition is carried out using deep maxout network. The data augmented output *D*_1_ is presented as an input for the DMN classifier for recognizing the emotions. This classifier is also trained by the proposed FASSO approach for the effective recognition. The DMN classifier [9] is a trainable activation function built in a multilayer structural arrangement. The major benefit of using this classifier is that it can efficiently improve the speed of the training process. Here, the input *D*_1_ is subjected into a network and the activation function of the hidden unit is expressed as follows:

(8) $$\begin{array}{c} f_{j,i}^{1}=\underset{i\in \left[1,e_{1}\right]}{\max}y^{T}L_{ji}+a_{ji},\\ f_{j,i}^{2}=\underset{i\in \left[1,e_{2}\right]}{\max}{f_{j,i}^{1}}^{T}L_{ji}+a_{ji},\\ f_{j,i}^{n}=\underset{i\in \left[1,e_{n}\right]}{\max}{f_{j,i}^{n-1}}^{T}L_{ji}+a_{ji},\\ f_{j,i}^{m}=\underset{i\in \left[1,e_{m}\right]}{\max}{f_{j,i}^{m-1}}^{T}L_{ji}+a_{ji},\\ g_{j}=\underset{i\in \left[1,e_{m}\right]}{\max}f_{j,i}^{m}, \end{array}$$

where *e*_*n*_ represents the overall units in the *n*^th^ layer and *m* signifies the total layers in the DMN classifier. The conventional nonlinear activation functions, like absolute value rectifier and rectified linear, can be approximated effectively by DMN. The arbitrary activation functions would be approximated by the deep maxout network by maximizing the factors even in a complex nonlinear activation function. Thus, the classified output obtained is denoted as *R*.  Figure 2 illustrates the structure of DMN classifier.

#### 3.2.1. Training Procedure of Deep Maxout Network Using FASSO

The training of the DMN is carried out by the proposed FASSO algorithm and thereby obtaining an optimal solution. In fact, the FASSO algorithm is designed by the hybridization of FAT [21] and SSOA [23]. FAT is motivated from the transportation of the organic matters and revised ideas of branches. Here, organic matter transportation is designed with feedback approach of moistures. The exchange process of the entire material means that both the organic matter transfer and the moisture feedback are taken into consideration. FAT is very efficient in solving different kinds of optimization problems and it can adaptively handle the parameter for enhancing the search efficiency. On the other hand, SSOA is motivated by the behavior of the shepherd. Here, the agents are partitioned into multicommunities and the optimization procedure is carried out based on shepherd characteristics in nature working on every community. The proposed FASSO method exhibited robust performance and achieved better optimal solution. By incorporating the FAT with the SSOA, the optimization complexities are reduced in an effective way. The following are the algorithmic phases of the proposed FASSO model.

(i) Initialization: The branch population is initialized with *Z* number of branches and is given as follows:
  (9) $$\begin{array}{c} Y=\left\{Y_{1},Y_{2},\cdots ,Y_{w},\cdots ,Y_{Z}\right\};1\leq w\leq Z, \end{array}$$

where *Z* denotes the total branches and *Y*_*w*_ signifies *w*^th^ branch.

(ii) Compute fitness measure: The fitness measure is used to compute the optimal solution by calculating the optimal fitness value, and the fitness measure equation is formulated as follows:

(10) $$\begin{array}{c} \text{Fitness}=\frac{1}{U}\overset{U}{\underset{\rho =1}{\sum }}{\left[R-O_{\tau }\right]}^{2}, \end{array}$$

where *R* indicates the classifier output and *O*_*τ*_ represents the target output.

(iii) Update solution: Once the fitness value is computed, the update solution is achieved using the proposed FASSO approach. According to FAT algorithm, the update solution of the self-evolution operator is given as follows:

(11) $$\begin{array}{c} Y_{\text{new}}=Y_{i}+\mathrm{rand}\left(0,1\right)\left(Y_{\text{best}}-Y_{i}\right), \end{array}$$

(12) $$\begin{array}{c} Y_{\text{new}}=Y_{i}+\mathrm{rand}\left(0,1\right)Y_{\text{best}}-\mathrm{rand}\left(0,1\right)Y_{i}, \end{array}$$

(13) $$\begin{array}{c} Y_{\text{new}}=Y_{i}\left(1-\mathrm{rand}\left(0,1\right)\right)+\mathrm{rand}\left(0,1\right)Y_{\text{best}}. \end{array}$$

To solve the optimization issues and to improve the algorithm performance, FAT algorithm can be incorporated with FA, and thus, the standard equation of SSOA is as follows:

(14) $$\begin{array}{c} Y_{i}^{\text{temple}}=Y_{i}^{\text{old}}+\text{stepsize}, \end{array}$$

(15) $$\begin{array}{c} Y_{i}^{\text{temple}}=Y_{i}+\delta \mathrm{rand}\circ \left(Y_{c}-Y_{i}\right)+\beta \mathrm{rand}\circ \left(Y_{d}-Y_{i}\right), \end{array}$$

(16) $$\begin{array}{c} Y_{i}^{\text{temple}}=Y_{i}+\delta \mathrm{rand}\circ Y_{c}-\delta \mathrm{rand}\circ Y_{i}+\beta \mathrm{rand}\circ Y_{d}-\beta \mathrm{rand}\circ Y_{i}, \end{array}$$

(17) $$\begin{array}{c} Y_{i}^{\text{temple}}=Y_{i}\left(1-\delta \mathrm{rand}-\beta {\mathrm{rand}}_{i}\right)+\delta \mathrm{rand}\circ Y_{c}-\beta \mathrm{rand}\circ Y_{d}, \end{array}$$

(18) $$\begin{array}{c} Y_{i}=\frac{Y_{i}^{\text{temple}}-\delta \mathrm{rand}\circ Y_{c}-\beta \mathrm{rand}\circ Y_{d}}{1-\delta \mathrm{rand}-\beta \mathrm{rand}}. \end{array}$$

Substituting Equation (18) in Equation (13),

(19) $$\begin{array}{c} Y_{\text{new}}=\frac{Y_{\text{new}}-\delta \mathrm{rand}\circ Y_{c}-\beta \mathrm{rand}\circ Y_{d}}{1-\delta \mathrm{rand}-\beta \mathrm{rand}}\left(1-\mathrm{rand}\left(0,1\right)\right)+\mathrm{rand}\left(0,1\right)Y_{\text{best}},\\ Y_{\text{new}}=\frac{Y_{\text{new}}}{1-\mathrm{rand}\left(\beta +\delta \right)}\left(1-\mathrm{rand}\left(0,1\right)\right)-\frac{\delta \mathrm{rand}\circ Y_{c}-\beta \mathrm{rand}\circ Y_{d}}{1-\mathrm{rand}\left(\beta +\delta \right)}\left(1-\mathrm{rand}\left(0,1\right)\right)+\mathrm{rand}\left(0,1\right)Y_{\text{best}},\\ Y_{\text{new}}-\frac{Y_{\text{new}}}{1-\mathrm{rand}\left(\beta +\delta \right)}\left(1-\mathrm{rand}\left(0,1\right)\right)=\mathrm{rand}\left(0,1\right)Y_{\text{best}}-\frac{\delta \mathrm{rand}\circ Y_{c}-\beta \mathrm{rand}\circ Y_{d}}{1-\mathrm{rand}\left(\beta +\delta \right)}\left(1-\mathrm{rand}\left(0,1\right)\right),\\ Y_{\text{new}}\left(1-\frac{1-\mathrm{rand}\left(0,1\right)}{1-\mathrm{rand}\left(\beta +\delta \right)}\right)=\mathrm{rand}\left(0,1\right)Y_{\text{best}}-\frac{\delta \mathrm{rand}\circ Y_{c}-\beta \mathrm{rand}\circ Y_{d}}{1-\mathrm{rand}\left(\beta +\delta \right)}\left(1-\mathrm{rand}\left(0,1\right)\right),\\ Y_{\text{new}}\left(\frac{1-\mathrm{rand}\left(\beta +\delta \right)-1+\mathrm{rand}\left(0,1\right)}{1-\mathrm{rand}\left(\beta +\delta \right)}\right)=\mathrm{rand}\left(0,1\right)Y_{\text{best}}-\frac{\delta \mathrm{rand}\circ Y_{c}-\beta \mathrm{rand}\circ Y_{d}}{1-\mathrm{rand}\left(\beta +\delta \right)}\left(1-\mathrm{rand}\left(0,1\right)\right),\\ Y_{\text{new}}=\frac{1-\mathrm{rand}\left(\beta +\delta \right)}{\mathrm{rand}\left(0,1\right)-\mathrm{rand}\left(\beta +\delta \right)}\left[\mathrm{rand}\left(0,1\right)Y_{\text{best}}-\frac{\delta \mathrm{rand}\circ Y_{c}-\beta \mathrm{rand}\circ Y_{d}}{1-\mathrm{rand}\left(\beta +\delta \right)}\right],\\ \beta =\beta_{0}-\frac{\beta_{0}}{\varpi }\times \phi ,\\ \delta =\delta_{0}+\frac{\delta_{\max}-\delta_{0}}{\varpi }\times \phi , \end{array}$$

where *Y*_best_ signifies the best position in branch, rand lies between the range [0, 1], *w* denotes the maximum iterations, *ϕ* represents the iterations, and *Y*_*c*_, *Y*_*d*_ indicates the solution vectors of chosen horse and chosen sheep.

(iv) Feasibility evaluation: The feasibility evaluation is performed to obtain the optimal value with respect to the fitness function. If the newly achieved solution has the optimal value than the existing one, then the existing solution is replaced with newly obtained optimal value.
(v) Termination: All the abovementioned steps are repeated until best solution is achieved.

The pseudo code of the proposed FASSO algorithm is as follows.

## 4. Results and Discussion

**Figure 3:**
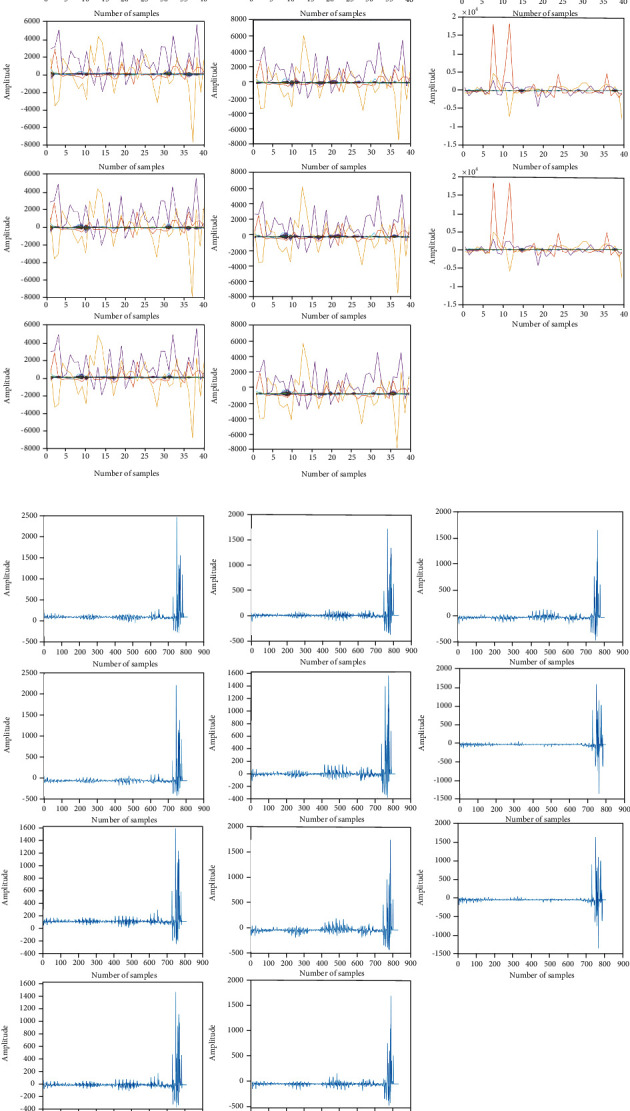
Experimental outcomes: (a) input EEG signal and (b) DWT feature output.

The experiment of the proposed FASSO-driven deep maxout network technique is conducted using the DEAP dataset [25]. The implementation is carried out in a MATLAB environment.  Figure 3 illustrates the experimental outcomes of the proposed method. The input EEG signals are shown in Figure 3(a), and the DWT features for the corresponding input signals are depicted in Figure 3(b). The EEG input and feature output signal are plotted with amplitude along the *y*-axis and number of samples along the *x*-axis. Each graph shows variation in the amplitude range and the corresponding extracted DWT features.

The performance evaluation of the proposed algorithm is carried out based on three metrics, namely, accuracy, sensitivity, and specificity.

- Accuracy: It is used for computing true positive and the true negative results of the recognized emotions.

(20) $$\begin{array}{c} \text{Accuracy}=\frac{T^{p}p+T^{n}}{T^{p}+F^{n}+F^{p}+F^{n}}, \end{array}$$

where *T*^*p*^is true positive value, true negative is *T*^*n*^, false positive value is *F*^*p*^, and false negative value is *F*^*n*^..

(ii) Specificity: It is a measure utilized for calculating the exact true negative outcomes of the recognized emotions.

(21) $$\begin{array}{c} \text{Specificity}=\frac{T^{n}}{T^{n}+F^{p}}. \end{array}$$

(iii) Sensitivity: It is used to compute the true positive values of the recognized emotions.

(22) $$\begin{array}{c} \text{Sensitivity}=\frac{T^{p}}{T^{p}+F^{n}}. \end{array}$$

The experimental outcomes of the proposed FASSO-based deep maxout network are illustrated in Figure 3. The input EEG signal and the corresponding DWT feature output for the first set of samples are shown in Figures 3(a) and 3(b), respectively. Similarly, Figures 3(c) and 3(d) depict the input EEG signal and the corresponding DWT feature output.

### 4.1. Performance Analysis

The performance evaluation of the proposed FASSO-based deep maxout network is carried out by varying the percentage of training data and the number of iterations.

#### 4.1.1. Performance Analysis Based on Training Data Percentage

**Figure 4:**
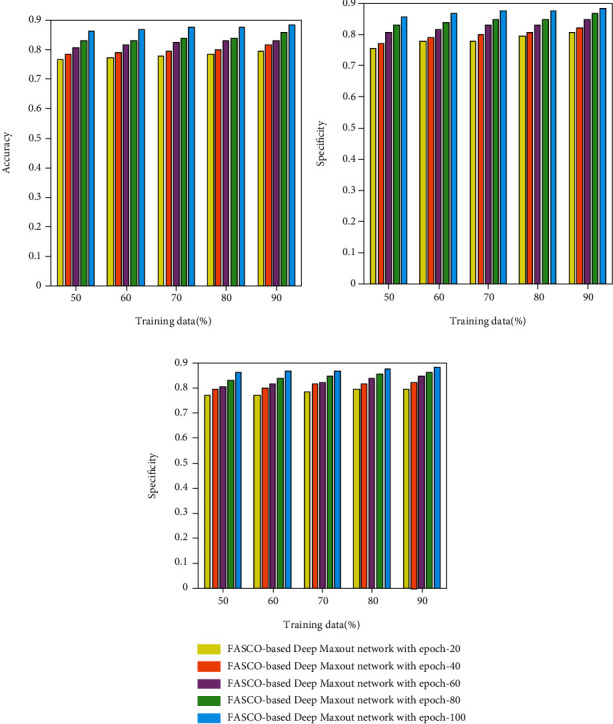
Performance assessment of FASSO-based deep maxout network using training data: (a) accuracy, (b) specificity, and (c) sensitivity.

The performance assessments of the proposed method with respect to accuracy, specificity, and sensitivity are illustrated in Figure 4 by varying the iterations for different values. Figures 4(a), 4(b), and 4(c) depict the performance analysis of the proposed method with respect to accuracy, specificity, and sensitivity. The accuracy value obtained by the FASSO-based deep maxout network with an epoch values of 20, 40, 60, 80, and 100 is 0.771, 0.791, 0.817, 0.830, and 0.865, respectively, for the 60% training data. Similarly for the 50% training data, the specificity value achieved by the proposed method with epoch 20 is 0.755, epoch 40 is 0.771, epoch 60 is 0.806, epoch 80 is 0.829, and epoch 100 is 0.855. Similarly, by considering the training data as 70%, the sensitivity metrics of the proposed method for an epoch 20 is 0.763, epoch 40 is 0.783, epoch 60 is 0.792, epoch 80 is 0.798, and epoch 100 is 0.842.

#### 4.1.2. Comparative Analysis

This section illustrates the comparison of the proposed FASSO-based DMN based on training data and *K*-fold. The comparative analysis is carried out with the existing methods, namely, regularized graph NN [5], LSTM [10], RNN+ensemble [12], and CNN+LSTM [15], deep maxout network, FAT-based deep maxout network, and SSOA-based deep maxout network (Table 1).

**Figure 5:**
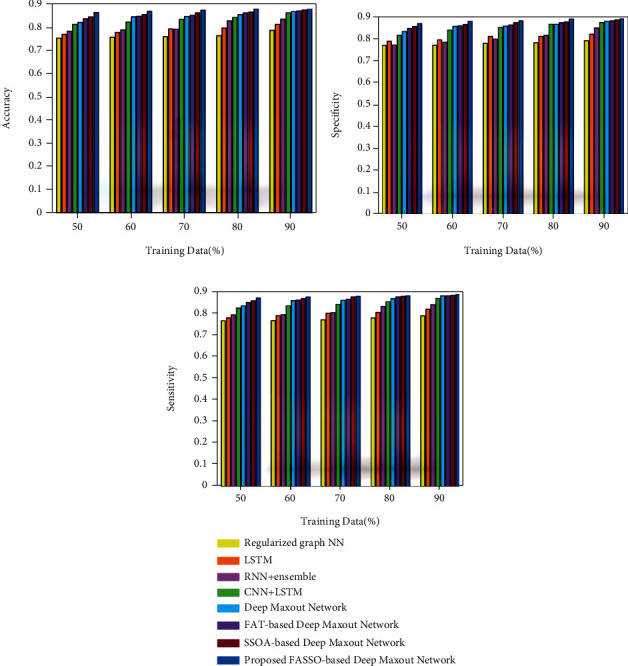
Assessment using training data: (a) accuracy, (b) specificity, and (c) sensitivity.

Analysis using training data: Figure 5 illustrates the comparative assessment of developed FASSO-based deep maxout network based on the metrics, such as accuracy, specificity, and sensitivity by considering the training data percentage.  Figure 5(a) illustrates the accuracy assessment. The accuracy values obtained by the techniques, such as regularized graph NN, LSTM, RNN+ensemble, CNN+LSTM, deep maxout network, FAT-based deep maxout network, SSOA-based deep maxout network, and the proposed FASSO-based deep maxout network, are 0.759, 0.780, 0.791, 0.824, 0.847, 0.849, 0.856, and 0.871 for the training data 60%. The performance improvement achieved by the developed FASSO-based deep maxout network technique in comparison with the existing techniques is 12.80%, 10.40%, 9.138%, 5.327%, 2.76%, 2.53%, and 1.72%, respectively.

The analysis using specificity metric is presented in Figure 5(b). The proposed FASSO-based deep maxout network measured a specificity value of 0.889, while the specificity values achieved by the existing techniques are 0.782, 0.811, 0.815, 0.865, 0.866, 0.874, and 0.877, respectively, for the training data 80%. The performance gains computed by the proposed method in comparison with the existing techniques are 12.08%, 8.80%, 8.353%, 2.686%, 2.59%, 1.69%, and 1.35%, respectively.  Figure 5(c) presents the assessment based on the sensitivity metric. By considering the training data as 70%, the sensitivity value achieved by the proposed method is 0.879, while the sensitivity values obtained by the existing techniques are 0.771, 0.801, 0.804, 0.841, 0.861, 0.865, and 0.877. Moreover, the performance improvements measured by the proposed FASSO-based deep maxout network by comparing with the existing methods are 12.34%, 8.956%, 8.604%, 4.387%, 2.05%, 1.59%, and 0.23%.

**Figure 6:**
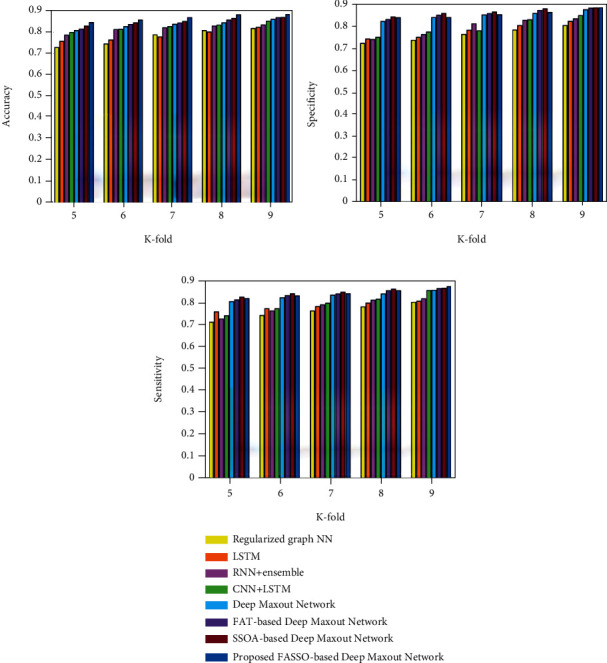
Assessment using *K*-fold: (a) accuracy, (b) specificity, and (c) sensitivity.

Analysis using *K*-fold: The comparative analysis of developed FASSO-driven deep maxout network based on the *K*-fold value with respect to the metrics such as accuracy, specificity, and sensitivity is presented in Figure 6.  Figure 6(a) portrays the assessment of accuracy metric. When *K*-fold value is 5, the accuracy value achieved by the developed FASSO-based deep maxout network is 0.845, while the accuracy values obtained by the techniques are as follows: regularized graph NN is 0.728, LSTM is 0.756, RNN+ensemble is 0.786, CNN+LSTM is 0.798, deep maxout network is 0.808, FAT-based deep maxout network is 0.815, and SSOA-based deep maxout network is 0.828. Moreover, the performance improvements measured by the proposed FASSO-based deep maxout network by comparing with the existing methods are 13.79%, 10.47%, 6.923%, 5.514%, 4.38%, 3.55%, and 2.01%.

The assessment based on specificity metric is presented in Figure 6(b). For the *K*-fold value is 9, the specificity values of the proposed FASSO-based deep maxout network are 0.88 and existing methods are 0.799, 0.819, 0.83, 0.844, 0.871, 0.878, and 0.879, respectively. The performance improvements achieved by the developed technique in comparison with the existing techniques are 9.2%, 6.93%, 5.68%, 4.09%, 1.02%, 0.113%, and 0.113%. The analysis using sensitivity is presented in Figure 6(c).

The proposed FASSO-based deep maxout network measured a sensitivity value of 0.875, while the sensitivity values achieved by the existing techniques are 0.804, 0.808, 0.819, 0.857, 0.859, 0.866, and 0.868, respectively, for the *K*-fold value 9. The performance gains computed by the developed FASSO-based deep maxout network in comparison with the existing techniques are 8.11%, 7.66%, 6.4%, 2.06%, 1.83%, 1.03%, and 0.8%.

### 4.2. Analysis Based on Computational Cost

The analysis based on the computational cost is provided in Table 2. The proposed FASSO-based deep maxout network has the computational time of 0.0189654 sec, which is the minimum computational time as compared to other methods. Here, the regularized graph NN has the high computational time of 0.634567 sec.

## 5. Conclusion

An efficient and robust FASSO-based deep maxout network classifier system is developed and its performance is evaluated using EEG signals. The proposed method is designed by combining FAT and SSOA algorithms. The experiment is conducted using DEAP database and its performance is evaluated with various performance metrics. The proposed method is also compared with the existing techniques to validate the obtained results. The improved outcomes in terms of accuracy, specificity, and sensitivity show the effectiveness of the proposed algorithm. The future enhancement of the proposed work would be the reflection of various deep learning classifiers for an accurate recognition and classification of human emotions. Also, the training process can be further improved by incorporating various optimization algorithms.

## Figures and Tables

**Algorithm 1:**
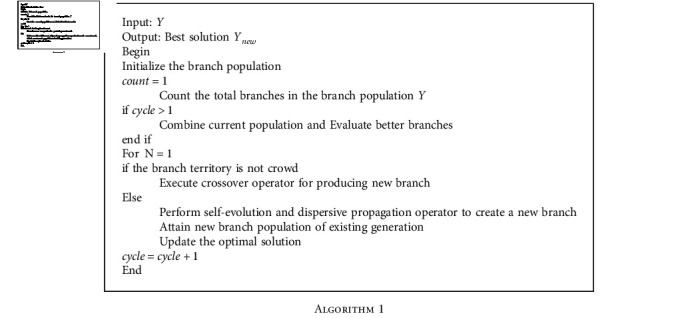


**Table 1:** Comparison of the proposed method with the existing techniques using training data and the *k*-fold.

Data type	Metrics	Regularized graph NN [5]	LSTM [10]	RNN+ensemble [12]	CNN+LSTM [15]	Deep maxout network	FAT-based DMN	SSOA-based DMN	Proposed FASSO-based DMN
Using training data (%)	Accuracy	0.789	0.815	0.837	0.864	0.869	0.873	0.877	0.889
	Specificity	0.791	0.820	0.849	0.874	0.879	0.882	0.886	0.89
	Sensitivity	0.789	0.819	0.839	0.869	0.882	0.882	0.884	0.886
Using *K*-fold	Accuracy	0.817	0.822	0.833	0.851	0.859	0.868	0.869	0.882
	Specificity	0.799	0.819	0.83	0.844	0.871	0.878	0.879	0.880
	Sensitivity	0.804	0.807	0.818	0.857	0.859	0.866	0.868	0.874

**Table 2:** Computational cost.

Sl. no.	Methods	Computational time (sec.)
1	Regularized graph NN	0.634567
2	LSTM	0.334216
3	RNN+ensemble	0.2318095
4	CNN+LSTM	1.0306644
5	Deep maxout network	0.0337350
6	FAT-based deep maxout network	0.0289145
7	SSOA-based deep maxout network	0.0214567
8	Proposed FASSO-based deep maxout network	0.0189654

## Data Availability

DEAP dataset [24] is a dataset, which is used for analyzing the emotions using physiological, video, and EEG signals. Here, the peripheral physiological and the EEG signals of the 32 participants are recorded, and then, each video is rated by every participant with respect to the levels of arousal, dominance, valence, familiarity, like, and dislike. Among 32 participants, video of the frontal face is recorded for the 22 participants. Moreover, the input size of each data in the file and the label size are in such a way that every class is processed effectively.
